# The emergence of a global right to health norm – the unresolved case of universal access to quality emergency obstetric care

**DOI:** 10.1186/1472-698X-14-4

**Published:** 2014-02-27

**Authors:** Rachel Hammonds, Gorik Ooms

**Affiliations:** 1 Department of Public Health, Institute of Tropical Medicine, 155 Nationalestraat, Antwerp 2000, Belgium; 2 Law and Development Research Group, University of Antwerp, Faculty of Law, Venusstraat 23, Antwerp 2000, Belgium

## Abstract

**Background:**

The global response to HIV suggests the potential of an emergent global right to health norm, embracing shared global responsibility for health, to assist policy communities in framing the obligations of the domestic state and the international community. Our research explores the extent to which this global right to health norm has influenced the global policy process around maternal health rights, with a focus on universal access to emergency obstetric care.

**Methods:**

In examining the extent to which arguments stemming from a global right to health norm have been successful in advancing international policy on universal access to emergency obstetric care, we looked at the period from 1985 to 2013 period. We adopted a qualitative case study approach applying a process-tracing methodology using multiple data sources, including an extensive literature review and limited key informant interviews to analyse the international policy agenda setting process surrounding maternal health rights, focusing on emergency obstetric care. We applied John Kingdon's public policy agenda setting streams model to analyse our data.

**Results:**

Kingdon’s model suggests that to succeed as a mobilising norm, the right to health could work if it can help bring the problem, policy and political streams together, as it did with access to AIDS treatment. Our analysis suggests that despite a normative grounding in the right to health, prioritisation of the specific maternal health entitlements remains fragmented.

**Conclusions:**

Despite United Nations recognition of maternal mortality as a human rights issue, the relevant policy communities have not yet managed to shift the policy agenda to prioritise the global right to health norm of shared responsibility for realising access to emergency obstetric care. The experience of HIV advocates in pushing for global solutions based on right to health principles, including participation, solidarity and accountability; suggest potential avenues for utilising right to health based arguments to push for policy priority for universal access to emergency obstetric care in the post-2015 global agenda.

## Background

The international human rights system that emerged from the ashes of World War Two largely reflected the prevailing Westphalian norm of state sovereignty [1]^a^. This sovereignty norm holds that the obligation of sovereign states to realise rights exists within their borders. However, the seeds of a new norm of shared international responsibility can also be found in the various international human rights treaties that the vast majority of nations have ratified in the past seventy years. With respect to economic, social and cultural rights, article 2.1 of the International Covenant on Economic, Social and Cultural Rights (the Covenant), enshrines this norm of shared responsibility under which nation states commit to taking steps individually and through “international assistance and cooperation, especially economic and technical” to realise Covenant rights, including article 12 the right to the highest attainable state of physical and mental health [2]. This article examines this norm of shared responsibility for realising the right to health, what we shall term a global right to health norm [3]. We define the global right to health norm as affirming the existence of a shared responsibility to respect, protect and fulfil the right to health, which challenges the dominant norm of exclusive national self-reliance [4]. To be clear, the emerging global right to health norm asserts the primacy of the state as the key duty bearer, charged with realising rights on its territory, but adds what lawyers term the transnational or extraterritorial dimension, the obligations of the international community to ensure that health rights do not remain the privilege of a minority of the world’s population [5].

Progress on scaling up health interventions in low-income countries to narrow the health equity gap often requires technical and financial co-operation (bi-lateral and/or multi-lateral) with the international community, a shared commitment to realising health rights, exemplified by what we term the global right to health norm. The power of this emerging norm in mobilising a global response to addressing the HIV epidemic has been well-documented and researched [6,7]. The huge scale up of financing and roll out of AIDS treatment from 2001–2010 is one example of how the international community has worked with national governments in low-income highly affected countries to jointly implement an element of the shared obligation to fulfil the right to health; universal access to anti-retroviral treatment (ART) [8,9]. This is one reason the global response to HIV/AIDS is termed “exceptional” and is arguably evidence of the emerging global right to health norm in action [10]. Further, access to ART offers an example of how shared responsibility for realising the right to health can be approached; albeit with important caveats, including the limited focus on one disease.

The research and analysis reported in this paper focuses on an area in which this emerging global right to health norm has been arguably less successful to date, maternal health. Preventable maternal mortality and morbidity remain a glaring example of global inequity where the role of the international community in addressing these issues has been arguably less ambitious and more complex [11,12]. Recent World Health Organization research into the impact of human rights on maternal and child health highlights the importance of viewing health through a human rights lens. The study includes example from Brazil, Nepal, Malawi and Italy finding that “applying human rights to women’s and children’s health interventions not only helps governments comply with their binding obligations, but also contributes to improving the health of women and children [13]”.

The limited progress on achieving two key health related sexual and reproductive health Millennium Development Goals (MDGs), namely reducing maternal mortality by three quarters and providing universal access to reproductive health by 2015, is well-documented. The most recent evidence clarifies that of the approximately 287 000 women who die in pregnancy or childbirth annually, 99% of these deaths occur in developing countries and over half in sub-Saharan Africa alone [14]. The limited advances reflected in these figures are clear evidence of the impact of global health inequity which fatally undermines the human dignity of women and the prospects of building families and societies on principles of justice [15].

The majority of maternal deaths stem from four direct conditions; obstetric haemorrhage, hypertensive disorders, complications of unsafe abortion and puerperal related sepsis. All of these can be either largely prevented or managed by effective clinical interventions and in particular through access to quality emergency obstetric care (EmOC) [16]. Despite the strong evidence base testifying to its importance, EmOC has never garnered the attention, or the controversy, of other reproductive health issues like family planning or abortion. Further, evidence suggests that progress in prioritising and scaling up such an important intervention remains limited [17-20].

Our study focuses on progress in advancing universal access to quality EmOC because from a medical perspective it is a key evidence-supported intervention that significantly decreases maternal mortality and morbidity [21]. Additionally, from an international human rights law perspective it is an obligation of comparable priority to a core right to health obligation, requiring immediate action by national governments, and when necessary, the international community; giving rise to extraterritorial obligations of assistance [22]. As Lynn Freedman, of Columbia’s Averting Maternal Death and Disability Programme (AMDD) notes, “In a human rights analysis, EmOC is not just one good idea among many. It is an obligation [23]”.

The history of the limited progress on addressing maternal mortality as a shared responsibility at the global level reveals multiple causal factors. An important 2007 Lancet article by Jeremy Shiffman and Stephanie Smith, examining the role of the safe motherhood advocacy community in advancing global priority for maternal health, argued that difficulties relating to the actors and the nature of safe motherhood itself meant that safe motherhood was still in its infancy as a global health initiative. However, they concluded on an optimistic note that “2007 could present a window of opportunity to generate political support for the cause [24]”.

Since their study the United Nations Human Rights Council has recognised preventable maternal mortality as a human rights violation, a potentially important step towards the solidification of a new global right to health norm, specifically the shared responsibility for maternal health [25,26]. Given that discussions about maternal health rights now figure prominently in the global human rights community we decided to examine the role of the right to health in shaping political priority for maternal health [27,28]. We posit that the right to health, as an emerging global norm, has the potential to assist policy communities in framing the obligations of the domestic state and the international community and mobilizing political priority and funding, as it did with AIDS. Our research asks: if the global right to health is an emerging norm with the potential to assist policy communities, what role has it played in maternal health advocacy? By examining the global policy process around maternal health rights, with a focus on universal access to EmOC, our study seeks to contribute to explaining the role of the global right to health norm in attracting global policy priority and funding. We conclude by briefly examining how the global right to health norm could help shape the discussions about shared obligations for realising maternal health in the post-2015 global agenda.

## Methods

### Ethics statement

Our research protocol was approved by the Institutional Review Board of the Institute of Tropical Medicine, Antwerp, Belgium.

In examining why right to health based arguments may have been less successful in advancing universal access to maternal health services we chose to focus on universal access to EmOC, a key intervention for reducing maternal mortality and morbidity, and a fundamental reproductive rights issue [29]. Our analysis employs a right to health based approach because it provides a framework, international human rights conventions and treaties, clarifying the obligations of domestic states and the international community regarding health services as well as the services that all people are entitled to claim [30].

We adopted a qualitative case study approach applying a process-tracing methodology using multiple data sources to allow for a more complete picture of the policy agenda setting process [31].

We selected a case study approach because it is a methodology that is recognised as being well suited for studying interactions in a real life context [32]. Data collection involved an extensive literature review, including scholarly articles indexed in PubMed, academic articles and case law from the international and national human rights field and grey literature from civil society organizations, United Nations agencies and donors engaged in maternal health, sexual and/or reproductive health. We also participated in civil society (e.g. the EURONGOs annual conference in 2012) and donor agency led conferences (e.g. the European Union’s Development Days 2012).

To complement this research we conducted a limited number of in-depth, semi-structured interviews with key informants from the sexual and reproductive health community, the maternal health community (we loosely define this community as including those who focus on safe motherhood and maternal health as distinct from the more broadly based sexual and reproductive health and rights community that advocates for holistic, structural solutions) and academics. The interviewees were identified through the literature review and conferences. As our research aim was to better understanding specific issues we engaged in purposive samples of experts in the field we studied. Our aim was to speak with individuals with direct knowledge of the global level policy process with a preference for those who had both 20 years of experience and were still engaged with the issue. We chose to limit the sample size to a maximum of fifteen interviewees as the interviews were an additional, not the only, source of data and the expertise of those interviewed was high [33].

We contacted sixteen potential interviewees and conducted ten interviews. When permission was granted (eight interviews), the interviews were recorded and transcribed otherwise the answers were recorded manually (two interviews). We asked all interviewees common questions but focused our questions on their areas of expertise. The majority (seven) of the interviews were conducted by Skype or by telephone and the remainder were face to face (three). The interviews were conducted over a five month period.

To analyse our data we applied John Kingdon’s public policy agenda setting streams model which holds that three independent streams - problems, policies and politics - need to flow together for political priority to appear for a given issue [34]. We selected Kingdon’s approach to analysing the policy process because it helps to unpack the complexity surrounding how and why problems and policy solutions get onto the political agenda and how and why a problem’s ‘time comes’ [35].

This study is a purely qualitative study therefore qualitative-quantitative data triangulation of our findings was not possible. To address bias in our study we analysed data from two separate sources, the multi-disciplinary literature review and interviews with key informants from different stakeholders. As such limited data triangulation for confirmatory purposes was possible and our review suggests, but can not confirm, that our data is true and certain.

Our case study included the time period from 1985, the publication of a seminal article on maternal mortality by Alan Rosenfeld and Deborah Maine [36], until spring 2013. The time periods that we highlight in each stream overlap but are not identical because as Kingdon makes clear each stream in the policy process flows independently. Further the process of problem identification, developing solutions and the political process are non-linear and feed into one another. For an issue to make it onto the policy agenda the streams have to come together at a certain time and a political window needs to open for the issue to go through.

## Results

### The problem stream

This section addresses the emergence of maternal mortality as a human rights problem that is grounded broadly in international human rights law through an historical overview of key developments in its evolution from condition to problem. As we are interested in exploring where EmOC fits into this picture we highlight it where relevant.

Kingdon notes that “Conditions become defined as problems when we come to believe that we should do something about them” (p 109) [34]. Getting preventable maternal mortality on the global agenda was of differing priority to the two key overlapping advocacy and policy communities, namely the maternal health community and the sexual and reproductive health and rights community. Kingdon suggests that indicators, focusing events and feedback are key to this stage of the journey and our discussion highlights how each played a part.

#### The emergence of maternal mortality as a (global) human rights problem

Abou Zahr highlights that “Maternal mortality was a neglected issue during the 1970s and early 1980s, less because health professionals in developing countries were unaware of the problem than because they lacked the tools to quantify and analyse it [37]”. It was only in the mid-1980s that the World Health Organization (WHO) released its first estimates of the annual global maternal mortality death toll as exceeding half a million women per year, with the vast majority occurring in low-income countries [38]. The reporting of this indicator helped raise awareness of the scale of the problem and the global inequity it exposed [39]. It also became the basis of much advocacy and the over half a million annual deaths in childbirth or pregnancy was a figure that came to be associated with this problem until evidence of a decline was finally reported in 2010 [40].

Several key focusing events also helped push maternal mortality from a condition to a global problem. The 1985 Lancet article by Rosenfeld and Maine entitled “Maternal Mortality – a neglected tragedy. Where is the M in MCH?” played a big role in bringing the issue of preventable maternal mortality to the attention of the international health policy community [36]. Rosenfeld and Maine argued forcefully for increased attention to the mother, not just the child, and challenged the traditional focus on antenatal risk screening and traditional birth attendants presenting compelling arguments for the importance of access to EmOC; the significance of which we shall explore below.

A key focusing event in the emergence of a maternal health community was the 1987 launch of the global Safe Motherhood Initiative (SMI) in Kenya aiming to increase awareness of the over half a million annual maternal deaths and to reduce maternal mortality levels by half by 2000 [41]. The SMI launch was supported by the World Bank, WHO and United Nations Population Fund (UNFPA) and this broad based support meant the concept and name had to be acceptable for communities that opposed what they perceived as political or feminist implications of terms like reproductive health [24]. The important role of the SMI in shaping the current policy agenda on maternal health will be examined in the policy community section.

In parallel with these developments another key factor was the rise of the women’s movement which helped draw attention to the consequence of the global neglect of women’s human rights, including women’s health needs. A key focusing event included the landmark 1979 Convention on the Elimination of Discrimination against Women, which mainly framed women’s health in the context of reproductive health focusing on family planning [42]. The 1994 United Nations Cairo Conference on Population and Development (Cairo) and the follow-up 1995 Beijing World Conference on Women (Beijing) anchored sexual and reproductive rights as broader human rights claims [43]. With respect to maternal health governments made a key commitment at Cairo to reduce maternal mortality by one half of the 1990 levels by the year 2000 and half again by 2015 [44]. As Alicia Yamin notes the language of Cairo and Beijing went beyond focusing on women’s health and maternal health as simply issues of health and biology recognising that it is a matter of power relations [45]. For example the Beijing Platform recognised that “A major barrier for women to the achievement of the highest attainable standard of health is inequality; both between men and women and among women in different geographical regions, social classes and indigenous and ethnic groups [46]”. Thus while maternal health and maternal mortality remained important issues, for the sexual and reproductive health and rights movement they were not the priority, rather broader based holistic goals addressing structural impediments to realising rights were the focus.

The international human rights response to these developments further clarified the different human rights obligations of states with respect to maternal mortality. In 1999 the Committee on the Elimination of Discrimination against Women, issued General Recommendation 24 proclaiming “it is the duty of States parties to ensure women's right to safe motherhood and emergency obstetric services and they should allocate to these services the maximum extent of available resources [47]”.

The 2000 General Comment on the right to the health (General Comment) clarified the nature of the right to health obligations enshrined in Article 12 of the Covenant [2,30]. It added precision to the right to health dimensions of sexual and reproductive health further bringing attention to maternal health as a rights issue. It makes clear that the Covenant articles on women’s health require measures to improve sexual and reproductive health services… including access to emergency obstetric services (paragraph 14)^b^[30]. The framing of maternal health as an obligation of comparable priority to a core obligation has important funding and priority setting implications for the national government and the international community; specifically that states in a position to assist must help those that cannot fulfil their core maternal health obligations, including access to EmOC. The work of the United Nations Special Rapporteurs on the Right to Health; Paul Hunt (2002–2009) and Anad Grover (2009-present), has helped to keep maternal mortality high on the international human rights agenda [48].

It can be argued that maternal mortality moved from a condition to a global health problem when its reduction was included in the MDGs, as agreed in 2001. However its inclusion was a double edged sword. It exposed the tension around the framing of the issue among the different policy communities while further raising global awareness of the problem, if not significantly increased funding. For the maternal health community ‘their issue’ was on the global agenda which was a victory, albeit the indicator was not one they would have chosen. Much of the sexual and reproductive health and rights community were appalled that the Millennium Declaration omitted any language about sexual and reproductive rights, viewing the MDG agenda as undermining the progress made in Cairo and Beijing [49]. The late addition of MDG target 5B in 2005 relating to universal access to reproductive health, including family planning was received more positively by the sexual and reproductive health and rights community.

It is also important to note that although maternal mortality was now being framed as a human rights problem by diverse policy and advocacy communities, each community’s understanding of and approach to addressing this frame was different. As one interviewee noted, “*The people in maternal and child health are not human rights advocates so they do not have the tools for such advocacy.”* (Interview 8) For the sexual and reproductive health and rights community, maternal mortality was a reproductive health rights issue requiring a broad based structural response addressing gender discrimination and the power imbalances within and between countries. For the maternal health community maternal mortality was primarily a health issue requiring the appropriate medical interventions guaranteed in a well-functioning health system. This overlapping, but increasingly disparate framing of their agendas led to fragmentation in the policy community as shall be discussed below.

2009 saw a landmark resolution adopted by the United Nations Human Rights Council recognizing maternal mortality as a human rights violation and follow-up resolutions and work led to the 2012 UN Technical Guidance on Preventable Maternal Mortality and Morbidity and Human Rights, (Technical Guidance) which will be examined below [50]. It provides operational guidelines on program design and policy implementation that are consistent with human rights standards as well as highlighting the importance of accountability mechanisms and the role of the international community in addressing maternal mortality and morbidity.

To summarize; only since the new millennium, and the publication of General Comment 14, has the international human rights framework developed sufficiently clear guidance with respect to health related obligations and priority setting for national governments and the assistance and cooperation obligations for the international community (extra-territorial obligations), although clearly more work is needed^b^. Ongoing work to further clarify the implications of health related (and other) extra-territorial obligations will improve specificity but for the purposes of the main issue covered in our study, namely access to EmOC, there is sufficient legal guidance for national and international policy makers to act to fulfil their shared obligations [51].

### The policy stream

In this section we analyse the policy stream around maternal mortality to see how it evolved, maintaining a focus on access to EmOC. As noted in the section on the problem stream this is a complex field with multiple players each with a distinct but overlapping agenda, constituency and approach to achieving their goals; potentially leading to fragmentation.

In Kingdon’s model the policy stream is the second element of the policy process producing the alternative solutions to address the problem. In our case the primary actors are the two main policy communities who work with different technical experts, academics, international agencies, national governments and donors to develop solutions that will help reduce maternal mortality globally. It is at this stage in the policy process that proposals are generated, debated, reworked and eventually accepted or rejected. Kingdon notes that the policy communities involved in this process can be tight knit or fragmented, the consequences of fragmentation being disjointed policy, lack of common orientation and agenda instability [34]. He terms the mix of ideas from which solutions arise the “policy primeval soup”.

In analysing the policy soup we will use universal access to EmOC as a tracer for the right to health approach. We focus on access to EmOC because it a key evidence based intervention vital for reducing maternal mortality in all countries and it is identified as an obligation of comparable priority to a core obligation under the right to health.

On the technical side, health systems policy specialists argue that they know what needs to be done to scale up access to EmOC, while acknowledging that it is highly complex and context specific. A first step in making it a reality is generating both political commitment and funding (domestic and international funding). Before a policy community can advance its claim for funding in the political realm, Kingdon argues, it needs a solution to emerge from the policy soup.

#### Lack of consensus on a resonating framework in the different reproductive health communities – maternal health and sexual and reproductive health and rights

As Shiffman and Smith noted in their article in 2007 the maternal health community and the sexual and reproductive health and rights community could not articulate a common internal or external problem frame [24]. It seems obvious to state that reducing maternal mortality and morbidity is the key focus of the maternal health community. However it is worth highlighting because the sexual and reproductive health and rights community embraces a more holistic agenda that includes maternal health but not as a main focus. Although as discussed above both communities worked towards a common purpose in having maternal mortality understood and addressed as a human rights issue by the United Nations they have not worked on a common policy to address this issue. Sara Davies characterises the two groups as the right to reproductive health care group and the other as the right to reproductive self-determination group, noting that although they have a different strategy and focus they have both focused on the need for women’s health to be expressed as a right [52]. One respondent also suggested “*one point on why it has taken the maternal health people so long to use a human rights approach is that I think they have just been so pre-occupied with trying to establish the standards for improvement, for example for EmOC. First of all to know what the most important health intervention was, a lot of public health epidemiological work went into it”* (Interview 8).

Sexual and reproductive health and rights advocacy and solutions to reducing maternal mortality are more comprehensive, political and advocate structural change. This broader focus meant it has not put its full weight behind most maternal mortality reduction advocacy that adopts a less politicised safe motherhood type approach. In addition, it has championed other more political causes more closely tied to advancing the Beijing and Cairo agendas. Marge Berer and Sundari Ravandran observed that “After the 1994 ICPD, the newly agreed reproductive and sexual health agenda, with its equivocal paragraph on unsafe abortion, seemed for a time to have ‘displaced’ the Safe Motherhood agenda, or at least put into question the priority it was to be given [53]”. In addition, the conservative backlash to Beijing and Cairo led to sexual and reproductive health and rights advocates prioritising different fights [54].

Shiffman and Smith’s study confirms this analysis noting that the framing of maternal mortality as safe motherhood did not attract the support of the women’s movement [24]. Further, today it is not an issue that energises much of the sexual and reproductive health community. One of our respondents, stated that for their (sexual and reproductive health and rights) organisation focusing on maternal mortality was not a priority noting “*I think that a lot of people working in sexual and reproductive health think that maternal mortality should not be an issue anymore. We know we can’t have it happen and that you need to have the hospitals and the quality of care but the whole relationship issue, it is about power”* (Interview 1).

In recent years various members of the sexual and reproductive health and rights community, representing diverse groups from around the world, have argued for the need to re-politicise the sexual and reproductive health and rights agenda. For many, the limited MDG agenda reaffirmed a specific concern that since the gains of the Cairo and Beijing in the 1990s, reproductive health has been narrowed to safe motherhood or simply surviving pregnancy [54]. The 2010 World Bank reproductive health action plan 2010–2015 noted that “maternal health has not emerged as a political priority for a number of reasons and that the rise of competing priorities and the loss of focus on family planning within the broader ICPD agenda have contributed to declining attention and funding [55]”.

Since the mid-1990s the sexual and reproductive health and rights community’s prime focus is on advancing the holistic Cairo and Beijing principles and commitments, which is not aligned with the main focus narrower focus of the maternal health community [11]. The disparate focus of these groups means that EmOC never really makes it onto their common agenda. The maternal health community continues to focus on the three pillar solution advanced by the United Nations Population Fund (UNFPA); family planning, skilled attendance at birth and access to EmOC [56]. These important goals are less politically charged, and do not advocate the structural changes in power and gender dynamics underpinning Cairo and Beijing. As such, we suggest, it was easier for them to partner with other policy communities, including newborn and child health, and seize the opportunity of the open policy window in 2010, discussed below.

#### The maternal health community -multiple solutions and policy community fragmentation

Within the maternal health community disagreement over which medical intervention(s) to prioritise was problematic from the outset and initially hampered progress on advocating for a focused policy. In the late 1980s to 90s one school pushed for low-cost interventions for reducing maternal mortality that focused on predicting and preventing obstetric complications, clean birthing practices and training traditional birth attendants. This contrasted with those persuaded by the arguments in Maine and Rosenfeld’s 1985 article which argued forcefully for the need to scale up access to EmOC, stressing that it was not possible to adopt preventive strategies to address maternal mortality stemming from unpredictable obstetric causes [57]. The spectre of demanding what was perceived as a high cost intervention continues to cast a shadow over discussions [58,59]. It was gradually accepted that all pregnant women are at risk of life-threatening obstetric complications with each pregnancy and that screening does not work. This did not prevent further fragmentation as some advocates moved from advocating the training of skilled birth attendants and antenatal risk screening to the “skilled attendance approach [60,61]”.

As noted above both the Committee on Economic, Social and Cultural Rights (in General Comment 14) and the CEDAW Committee have recognized EmOC as a priority intervention [30,47]. The 1997 process indicators related to EmOC could be used as both public health and human rights indicators to drive policy and assess progress on implementation [23,62]. Despite being endorsed by key United Nations agencies including the UNFPA, WHO and UNICEF, vital funding commitments and implementation activities did not follow. The frustration of those advocating for EmOC to be prioritised comes through in a 2000 article by Maine in which she laments that the then newly issued WHO Safe Motherhood Needs Assessment.

“contains suggestions for evaluating (in this order): ‘ . . .policy on antenatal care services .. . policy on clean and safe delivery…policy on postpartum care for mother and newborn....policy on essential obstetric care . . . ’ The last item in this list includes the treatment of major complications. Why is ‘essential’ obstetric care (EOC) listed fourth? Why are the interventions which have been proven to save women’s lives listed after those whose value is questionable? [63]”.

Eventually the skilled attendance and EmOC approaches would be reconciled and included as complementary vital element in the three pillars of maternal mortality reduction as advanced by the UNFPA and supported by other global health actors requiring that:

1. All women have access to contraception to avoid unintended pregnancies.

2. All pregnant women have access to skilled care at the time of birth.

3. All those with complications have timely access to quality emergency obstetric care [64].

Of the three pillars of maternal mortality reduction EmOC has proved the hardest to gain traction. After a lengthy period of neglect mid 2012 saw family planning return to the top of the global agenda at the London Family Planning Summit co-sponsored by the UNFPA and the Bill and Melinda Gates Foundation [65]. The 2010 Lancet Commission Review of progress on the MDGs notes that “Except for financing initiatives initiated in very recent years, there is very little evidence of wide-scale interventions to increase the quantity or quality of, or the access to [skilled birth attendance]. Nor have credible efforts been made to improve access to Emergency Obstetric Care (EmOC) for women with complications. Rather, actions in support of MDG 5 often attempt to bypass a facility-based health system by seeking community-based solutions, such as educating women on warning signs of complications or training traditional birth attendants or community volunteers [66]”.

As Shiffman and Smith documented in their 2007 Lancet article the absence of agreement on which strategy to adopt was a key reason for the fragmentation of the safe motherhood (including much of the maternal health) policy community and its failure to generate sufficient international political priority from 1987–2006 [24].

Despite the widely accepted 1997 EmOC process indicators that allow for countries to track progress, the requisite political priority to fund EmOC scale up did not follow. A consequence is that ongoing measurement issues related to EmOC data quality or even absence in the countries most impacted by maternal mortality has proved an insurmountable obstacle in the competition for funding and priority that is even more target driven since the MDGs. As Yamin notes despite countries asking for improved access to EmOC with respect to commitments in the Global Strategy on Women and Children “the 2011 WHO Information and Accountability Commission report did not include EMOC among the eleven indicators (six related to maternal health) that it selected, precisely because they are not available in enough countries [67]”. A recent study on available facilities in six low coverage countries, Kenya, Malawi, Sierra Leone, Nigeria, Bangladesh and India concluded that availability of EmOC was well below minimum UN target coverage levels [68]. These UN targets are not included in the WHO Information and Accountability’ Commission’s (IAC) eleven indicators. In discussing the absence of EmOC indicators, like those found in the UN targets, one respondent stated, *“This policy stuff does not happen at local level. I think the responsibility is really at the international level and the importance of indicators is crucial. What you measure is what you report on”* (Interview 5).

#### The legacy of competition for donor attention and funding – the emergence of an integrated approach

The fact that newborn and child survival are intricately linked to maternal survival meant that newborn, child and the maternal health/safe motherhood communities have long competed for attention and funding [24,69]. In 2006 the global SMI joined with the Partnership for Maternal, Newborn and Child Health (PMNCH) which helped to bring these communities together^c^. Despite the uneasy nature of this union it has proved successful in helping attract political attention and funding for a new integrated, comprehensive policy.

The different members of the PMNCH have embraced the reproductive, maternal, newborn and child health continuum of care (RMNCH). The objective of this approach flows from the World Health Report 2005 and aims to offer a constellation of services and interventions for mothers and children from pre-pregnancy/adolescence, through pregnancy, childbirth and the postnatal/postpartum period, until children reach the age of five years [70]. One respondent remarking on the success of this approach noted that “*This was going to the solution to bring health systems approaches to the MDGs and accelerate progress to reduce the gaps etc.”* (Interview 2).

As we shall discuss later the PMNCH managed to push its approach through the political window that opened in mid-2009. A recent informal assessment of the RMNCH continuum of care by key thought and policy makers identified both positive and negative results. Starrs argues that “maternal health has not been marginalized within the continuum from a broad policy, program and funding perspective” citing evidence that of the 275 commitments to the Global Strategy 53% had maternal health content [71]. She noted that others, including Horton and Graham, welcomed the unifying impact of the RMNCH continuum but expressed concern that it may contribute to the compartmentalization of women and girls viewing them primarily as mothers or future mothers [71]. A respondent echoed this concern noting “*Now I think one important thing we now know is that the RNMCH continuum of care does not go far enough; we have talked about the impact of maternal mortality on family and society and what the RNMCH continuum does is an improvement but it does not go far enough”* (Interview 3).

### The political stream

The third stream in Kingdon’s policy process is the political stream. As his study focused on the policy process in a single country we have extrapolated from his wide-ranging analysis, extending from the importance of the national mood to the impact of the bureaucratic turf wars, to the international stage. As such we have been guided by studies on similar topics, including that of Shiffman and Smith, in which they suggested the need to examine international political developments and publicly visible actors like UN agency heads and the leaders of large advocacy organizations. In this section we focus on the MDGs and the recently established WHO IAC, key milestones and actors in maternal health.

#### Pressure to measure and show results

Since 2001 the MDGs have emerged as the dominant development assistance framework and in the area of health they have guided both national and international policy towards meeting health related targets. Although not legally binding, the target driven model has proved appealing to politicians and funders and consequently has pushed large international advocacy agencies to ensure they engage in actions that allow for reporting of tangible results. One respondent noted that their organisation, which focuses on sexual and reproductive health and rights, had previously eschewed targets due to the inherent difficulties of measuring key issues like empowerment. Since the MDGs they, and other partner organisations, have felt the need to embrace some of the more measurable outcomes to show donors the impact of their work, noting the following change, “*With maternal health you can focus on the numbers. You can easily say we need to decrease maternal mortality by X. That is what is attractive to donors. But that is very much a curative approach as opposed to a preventive approach.”* And continuing “*It is not that you focus on reproductive health and family planning that you should forget the rest. It is a selling point it is a way to get the interest and the funding of the donors. So that is a lesson learned”* (Interview 1)*.*

The problems inherent in using the globally established MDGs as national planning targets or for measuring progress have been the subject of considerable scholarship [72,73]. It is beyond the scope of this paper to assess how such target driven reporting impacts on policy and program planning beyond the obvious observation that they include measurement of a specific target, which focuses the attention of policy makers at global and national level. With respect to maternal mortality access to EmOC was neither an indicator nor a target.

From a right to health perspective it is clear that universal access to EmOC is an obligation that should be prioritised by both national and international actors, but the MDGs did not advance this. Further, as noted above the absence of EmOC among the eleven indicators included in the recent 2011 WHO IAC report is problematic for accountability and funding reasons, and more importantly, for the message it sends to countries with high maternal mortality rates as to which interventions to prioritise in addressing this issue.

#### Pressure to advance MDGs 4 and 5

Following the 2006 launch of the PNMCH the maternal health community hoped to be able to report positive news. A 2007 global estimate of maternal mortality showed that little progress had been made in decades. [74] This contrasted with progress on MDG 6. A 2010 study showed that the rise of vertical disease focused Global Health Initiatives, like the Global Fund to fight AIDS, Tuberculosis and Malaria (Global Fund) and the GAVI Alliance was accompanied by huge increases in funding to global health [9]. However from 1999–2008 the distribution of this funding was uneven, with a ten-fold increase in funding for communicable diseases like AIDS and tuberculosis and only a doubling of funds for maternal, newborn and child health.

In the lead up to the 2010 MDG summit it became clear that MDGs 4 (maternal mortality) and 5 (child mortality) were most off track and particularly in much of sub-Saharan Africa [40]. The maternal health community knew that a policy window would open at the MDG Summit in 2010.

### The joining of the streams

Kingdon’s model asserts that an issue emerges on a policy agenda when the problems, policies and politics stream couple. The continuum of care concept advanced by the PNMCH community offered a solution to reducing maternal mortality (MDG 5) and child mortality (MDG 4), two popular global political commitments. Political leaders welcomed the emergence of the PMNCH and its integrated agendas and the maternal, newborn and child health communities were able to harness political momentum building around this approach.

The MDG summit in 2010 was a perfect policy window for the PNMCH community to push its policy through and generate the necessary funding. It was here that United Nations’ Secretary-General Ban Ki-moon launched a ‘Global Strategy for Women’s and Children’s Health’ (Global Strategy) [75]. The Global Strategy embraced the continuum of care concept advanced by the PNMCH. One respondent commented, “*I would say, and I am not alone in this, Richard Horton said it too, the continuum of care reached its apotheosis in the Global Strategy”* (Interview 2).

The international community responded with the Canadians jumping on the bandwagon at the Toronto G8 summit and launching the Muskoka Initiative on Maternal Newborn and Under-Five Child Health, which included funding and accountability commitments [76]. International and national pledges to realize the Global Strategy were estimated at $40 billion between 2011 and 2015, or $8 billion per year [77]. Efforts to create an accountability structure led to the IAC [78]. The IAC recommended the establishment of both national level accountability mechanisms and a global independent Expert Review Group (iERG) mandated to review progress on IAC recommendations until 2015. As Yamin notes “this immense flurry of activities and commitments surpassed, by any measure, those made after the International Conference for Population and Development (Cairo) from funding to political commitments” (page 368) [67].

Where does support for policy to prioritize the scaling up EmOC stand after all of this? As noted above, it is not one of the key indicators used by the PNMCH or the iERG. One respondent noted “*Is there universal agreement that EmOC is a key pillar intervention for reducing maternal mortality and morbidity? Absolutely. Are there all kinds of problems in practice because it requires health systems interventions and not vertical interventions one off magic bullet solutions like this ridiculous shock suit? Or giving every trained birth attendant in the country misoprostol? Are there all kinds of problems with health work force and budgeting making sure that supply chains are working and there is a referral network. Yes. But I think there is absolute agreement at international and at national level that you need EmOC*” (Interview 2).

Some would argue that the failure of the maternal health community to persuade policy makers of the need to prioritize EmOC as an intervention is reflected in the recent Countdown to 2015 findings that “much remains to be done in the most crucial area – childbirth [79]”.

## Discussion

### Policy community fragmentation

The utility of applying Kingdon’s model to maternal health is that it helps us identify why maternal health rights policy has not succeeded in shifting from the dominant global norm of national self-reliance to the emerging GRH norm of shared obligations. Our limited analysis suggests that policy community fragmentation was a significant obstacle contributing to EmOC’s failure to make it onto the global political agenda. This fragmentation took two forms. First, although maternal mortality is now recognised as a human rights problem, the maternal health community and the sexual and reproductive health and rights community could not articulate a common external frame or unified demand, with respect to maternal health rights related obligations. Some may argue they took different but complementary approaches but from a policy perspective the slow rate of change suggests these different approaches have been an obstacle [80].

Neither of these communities robustly championed access to EmOC as a core right to health obligation that requires countries to take immediate steps to fulfil, and when necessary, claim international cooperation and assistance to assist them in fulfilling this goals. Although supportive of the rights approach the maternal health community focused on addressing maternal mortality as primarily a national, medical problem instead of using the power of the rights framework to push for obligations, like access to EmOC, to be fulfilled by national and global actors. In contrast, the sexual and reproductive health and rights community addressed maternal mortality, and access to EmOC as a symptom of a broader issue, worth addressing but not a focus of their work.

Second, there was also fragmentation with respect to alliances. The maternal health community pursued an alliance with the child and newborn health communities that depoliticized the maternal health agenda. With respect to maternal heath they focused on the three pillar solution but no pillar was prioritized. As these goals were less politically charged, not requiring the structural changes in power and gender dynamics underpinning Cairo and Beijing, they were not championed by the sexual and reproductive health and rights community. The maternal health community successfully partnered with other policy communities, including child health, and seized the opportunity of the open policy window and emerged with a role in the Global Strategy. However the entitlements included in this Global Strategy were governed by pragmatic, target driven concerns and did not address the more holistic rights driven agenda that would also involve more complex health systems solutions and a robust accountability mechanism to hold countries accountable for their commitments. This approach was not grounded in the right to health because although advocating for the three pillars it did not prioritise the core right to health related obligations of access to EmOC nor did address the issue of how to share global responsibility for realising rights. The sexual and reproductive health and rights community remained united behind the broad vision articulated in Cairo and Beijing and did not continue down this less politicized road.

### The HIV/AIDS experience

We can draw further lessons from the maternal health case study by contrasting it with the experience of AIDS activists in pushing the global community to support universal access to ART through their successful advocacy for shared responsibility for funding, creating an accountability mechanism and implementing policy [81]. In the 1980s and 1990s the life or death struggle against marginalization, discrimination and stigma in the American gay community was a key driver in transforming HIV/AIDS from a condition into a national problem. AIDS activists successfully engaged in vigorous advocacy that linked health outcomes and human rights deprivations. They advocated for big pharmaceutical companies to intensify research for treatment, a vaccine and a cure. Once a treatment was discovered they fought for it to be accessible for all in the United State and then they expanded their advocacy to push for access to treatment for all people; Americans, South Africans and Ethiopians. They based this advocacy on human rights principles drawing on the concepts of equity, universality and non-discrimination that underpin human rights and claiming that universal access to ART was an entitlement grounded in the right to health. As such they argued that if national governments could not afford the treatment the international community was obliged to assist. This appeal to international solidarity is a key element of what is termed AIDS exceptionality a response that embraced this concept from the outset and is encapsulated in the Harvard Consensus Statement, “AIDS treatment will always be more expensive than poor countries can afford; meaning that international aid is key to financing the effort [82]”.

So, AIDS activists had their global problem, universal access to ART, and a strong policy community that included civil society and the new agency, UNAIDS. They now needed a policy window to push their problem through.

The policy window they targeted was the 2000 G8 summit in Okinawa Japan and their efforts met success when, G8 leaders acknowledged the need for significantly greater resources to respond to AIDS, tuberculosis and malaria [83]. The momentum built and at a 2001 African Summit on AIDs UN Secretary General Koffi Annan called for the creation of a global trust fund to raise additional money. In June 2001, a UN General Assembly Special Session on AIDS committed to creating such a fund and by January 2002 the Global Fund permanent secretariat came into being [84].

A key reason that the Global Fund has been successful in ensuring that governments are held accountable for their funding commitments is its commitment to transparency [85]. Although the Global Fund and other global health initiatives have been criticized for their vertical approach to health interventions, the medium term funding model it operates is the type of longer term funding that is required for improving health systems, the type of funding needed to increase universal access to reproductive health services including EmOC [86]. As Lane and Glassman argue, *“[p]arts of the new institutional architecture, such as the Global Fund [to fight AIDS, Tuberculosis and Malaria], appear to deliver stable and predictable financing”*[87]. Attempts to expand the remit of the Global Fund to include health systems strengthening or maternal health have stalled.

### Moving forward

Kingdon’s model suggests that if the right to health operates as a global health norm if it can help bring the three streams (problem, policy and politics) together, as it did with access to treatment for HIV/AIDS. What lessons can policy and advocacy communities committed to women’s health rights (which includes both the maternal health community and the sexual and reproductive health and rights community) learn from the experience of the AIDS community? Our limited analysis suggests three key things, the power of rights to mobilise, the power of unity and the need for accountability.

#### Using the power of rights to mobilize

The recognition that maternal mortality is a human rights issue is a powerful construct whose potential to mobilize and empower people has not been fully explored. This suggest the importance of maternal health and sexual and reproductive health and rights advocates in further involving local communities in setting their advocacy priorities and using human rights strategies to shift the power to the disenfranchised. A recent study on rural Indonesia by Lucia D’Ambruoso et al. highlights the potential of a participatory tool like community audits to harness local knowledge and engagement to improve health planning and accountability in relation to EmOC [88].

One reason the AIDS community was able to maintain pressure on politicians was because of the power of affected communities demanding their rights be respected. At present it appears that those advocating for maternal health rights are less vocal and less well-organised and often the person whose rights have been violated and entitlements denied is dead. However increased advocacy by surviving family members suggests that communities are starting to claim their rights and asking for governments to be held accountable. Two landmark CEDAW cases involving Brazil and Peru reinforce the role international bodies can play in holding governments accountable for maternal deaths [89]. The experience of the AIDS movement suggests that to use the emergent global right to health norm effectively requires a more intensive engagement with local human rights experiences.

#### Using the new technical guidance to unify demands

As noted above the plurality of demands and approaches within the community advocating for maternal health and that focused on sexual and reproductive health and rights diminished the effectiveness of its response as its demands were fragmented. The maternal health community did not adopt a right to health mindset or approach – perhaps because they found the entitlements under the right to health insufficiently clear as to the appropriate health interventions. The sexual and reproductive health and rights demands were less focused on maternal health and more politically driven and thus arguably less attractive to those politicians and policy makers working within the current system. Perhaps the concise Technical Guidance recently adopted by the United Nations Human Rights Council (Technical Guidance) could provide a solution [90]. The operational guidance on implementing both policies and programs to reduce maternal mortality and morbidity is unique because unlike the Global Strategy it incorporates international human rights standards and advances the emerging GRH norm of shared responsibility (page 172) [45]. The Technical Guidance is firmly rooted in the broader Cairo and Beijing commitments and should thus help to operationalise more holistic strategies and garner broad based support. As such it offers the potential of a holistic approach to addressing maternal mortality as a multi-faceted problem that requires fundamental changes in way policies and programmes are designed and implemented, both within and beyond the health sector. Whether it can fulfil its promise is dependent on the support it receives from national governments, local communities and the international community; specifically with respect to funding. This brings us to our final point.

#### Using the right to health to focus demands for funding and accountability mechanisms

The AIDS movement made gains by demanding funding and mechanisms to track that funding and hold governments accountable rejecting what Yamin terms “failures of political will that are cloaked in claims of resource scarcity” [91]. Although the Global Strategy and the iERG are clearly positive steps several features suggest that with respect to maternal health the world has not yet progressed from a charitable approach to a GRH approach that entails shared obligations. First, the iERG has a limited term mandate (until 2015) and, unlike the Global Fund, is not firmly embedded in the global health governance structure. We agree with Shiffman that the creation of strong global institutions enhances the capacity to frame and negotiate issue portrayals which is vital for an issue’s sustainability on the global health agenda [92]. Past experience suggests that other global health actors engaged in maternal health, like UNFPA or WHO, will need to be strengthened and fully funded to ensure maternal health remains a priority past 2015. Thus in terms of post 2015 planning it is imperative that the iERG is transformed into or absorbed by a strong, well-funded global health institution. Second, the iERG has no way of ensuring its recommendations are respected. As such its oversight role does not build sufficiently on the current toothless country level periodic reviews conducted by the Committee on Economic, Social and Cultural Rights. As noted above a key strength of the Global Fund is its ability to hold partners and donors accountable. Maternal health requires a strong accountability mechanism to ensure national and international actors fulfil their human rights obligations.

Unfortunately the consequences of weak accountability are already showing with respect to countries honouring their funding pledges to the Global Strategy. A recent analysis of international assistance for maternal, newborn and child health from 2003 to 2010 by Hsu et al. showed that between 2009 and 2010 disbursements stagnated or slightly decreased for the first time since 2003 [93]. The first iERG report forcefully highlights this stating “Alarmingly, recipient countries describe reduced donor funding following the global financial crisis. These nations – the main concern of Every Woman, Every Child – reported jeopardised domestic financial flows because of global economic conditions. Only 20 of 49 countries have made financial commitments to the Global Strategy” (paragraph 19) [94]. In addition to this problem they mention an equally worrying point, “The iERG has no reliable data that provide an objective and quantitative assessment of the precise monies or promises committed and delivered… This absence of evidence is a major gap in the Global Strategy” (paragraph 20).

The Technical Guidance emphasizes that development partners have assistance and cooperation obligations (paragraphs 81–90), an expression of shared responsibility for realising global health rights, an affirmation of the GRH norm [95]. However in the absence of a well-funded, effective oversight mechanism that is respected by governments and civil society it will prove difficult to hold all actors accountable for their promises. The iERG report makes a similar point noting “countries should not expect the UN system, or its partner bodies, to deliver the Global Strategy without much greater and sustained investment to do so, including committed investment to ensure reliable, comprehensive, and independent measurement of progress” (paragraph 95). A recent announcement by World Bank President Jim Kim offers a possible solution, “… tonight I am announcing that the World Bank will establish a special funding mechanism to enable donors to scale up their funding to meet the urgent needs related to Millennium Development Goals 4 & 5. We hope to do this by leveraging the International Development Association (IDA), the World Bank’s fund for the poorest” [90].

## Conclusions

“The ideal that women should exercise free choice in maternity and survive pregnancy and childbirth is modest, but fundamental to the human dignity of women and to the building of families and societies on principles of justice [15]”.

In the lead up to 2015 Cook and Dickens’ “modest ideal” remains far from a reality. Analysis of our data suggests that despite a normative grounding in the right to health, the prioritization of the specific freedoms and entitlements related to maternal health have suffered from fragmentation at the policy level. This has impacted on the generation of political priority for addressing access to EmOC as a shared responsibility at the international level. This fragmentation may have delayed its place in the policy stream thus preventing it from becoming a priority on the global health agenda.

The formulation of a claim as a human right with corresponding obligations and entitlements enhances its weight in the global competition for scarce resources. Our study suggests that even though maternal mortality has been recognized as a rights issue the policy community has not yet managed to shift the normative agenda to embrace new rules addressing a key right to health obligation, shared responsibility for achieving universal access to EmOC. We remain cautiously optimistic that the Technical Guidance issued by the Human Rights Council may lead to fully funded and implemented national public health strategies that include health systems that meet the sexual and reproductive health needs of women [90].

As to whether they will garner similar international political and financial commitment as the UNAIDS/OHCHR guidelines for AIDS treatment remains an open question [96]. AIDS activists embraced and further developed the global right to health norm by advocating for a commitment to addressing health as a shared global responsibility, and not a responsibility confined solely to the borders and budget of a nation state. As noted above the Global Fund is a successful, albeit flawed, embodiment of shared responsibility. To replicate the success of the Global Fund in the field of maternal health will require upgrading the Global Strategy and the iERG. We suggest that the experience of HIV/AIDS advocates in pushing for global solutions based on human rights and right to health principles can offer useful tools to further advance respect for the fundamental rights vital for improving maternal health. Yamin argues that “Even the narrow issue of maternal mortality is not principally a medical problem; it is primarily a social problem and a problem of political will at both the national and political level” (page 177) [45]. The multiple challenges of the 21st century, in particular those of addressing climate change and sustainable development require a new approach to global governance. The transformative nature of the human rights agenda suggests that the time is ripe to look for global solutions based on human rights principles in combination with the SDGs. Health is not the only area in which rights based thinking offers new approaches. The United Nations Special Rapporteur on the right to food, Olivier De Schutter, has pressed governments to adopt food security strategies that empower women arguing, “Sharing power with women is a shortcut to reducing hunger and malnutrition, and is the single most effective step to realizing the right to food” [97]. We hope that the post-2015 agenda will be radical enough to satisfy Cook and Dickens’s “modest ideal” and unite all communities to fight for women’s health rights.

## Endnotes

^a^We employ Finnemore and Sikkink’s broad definition of a norm “as a standard of appropriate behaviour for actors with a given identity” [1].

^b^Paragraph 14 states “these may be understood as requiring measures to improve child and maternal health, sexual and reproductive health services, including access to family planning, pre- and post-natal care, **emergency obstetric services** and access to information, as well as to resources necessary to act on that information” [30] (emphasis added).

^c^One influential maternal health actor pushing for EmOC to be prioritised is Columbia University’s AMDD Program. Since 1999 it has helped to advance the case for EmOC within a human rights based approach advocating for countries to use the UN process indicators. However, although influential it is one voice of many.

## Competing interests

The authors declare that they have no competing interests.

## Authors’ contributions

RH and GO designed the research. RH conducted the interviews and drafted the manuscript. RH and GO revised the manuscript. Both RH and GO read and approved the final manuscript.

## Pre-publication history

The pre-publication history for this paper can be accessed here:

http://www.biomedcentral.com/1472-698X/14/4/prepub
